# Clinical Videoconferencing as eHealth: A Critical-Realist Review and Qualitative Meta-Synthesis

**DOI:** 10.2196/jmir.8497

**Published:** 2018-10-25

**Authors:** Anne Granstrøm Ekeland, Anne Helen Hansen, Trine Strand Bergmo

**Affiliations:** 1 Norwegian Centre for eHealth Research University Hospital of North Norway Tromsø Norway; 2 Department of Clinical Medicine Faculty of Health Sciences UiT The Arctic University of Norway Tromsø Norway; 3 Centre for Quality Improvement and Development University Hospital of North Norway Tromsø Norway; 4 Department of Community Medicine Faculty of Health Sciences UiT The Arctic University of Norway Tromsø Norway

**Keywords:** videoconferencing, clinical practice patterns, realist review, situated implications, eHealth, telemedicine

## Abstract

**Background:**

Earlier work has described videoconferencing technologies, peripheral equipment, organizational models, human relations, purposes, goals and roles as versatile, multifaceted, and those used differently in different clinical practices. Knowledge about benefits and challenges connected to specific characteristics of services are lacking. A 2005 systematic review of published definitions of electronic health (eHealth) identified 51 unique definitions. In addition, the “10 E’s of eHealth” was developed. In 2015, the question “What Is eHealth: Time for an Update?” was posed.

**Objective:**

Considering videoconferencing as eHealth, the objective of the paper is twofold: to demonstrate and cluster (different) clinical videoconferencing practices and their situated implications and to suggest interpretive concepts that apply to all clusters and contribute to generative learning of eHealth by discussing the concepts as add-ons to existing descriptions of eHealth in the “10 E’s of eHealth.”

**Methods:**

We performed a literature search via the National Center for Biotechnology Information, encompassing PubMed and PubMedCentral, for quality reviews and primary studies. We used the terms “videoconferencing” and “clinical practices.” The selection process was based upon clearly defined criteria. We used an electronic form to extract data. The analysis was inspired by critical and realist review types, grounded theory, and qualitative meta-synthesis.

**Results:**

The search returned 354 reviews and primary studies. This paper considered the primary studies, and 16 were included. We identified the following 4 broad clusters: videoconferencing as a controlled technological intervention within existing health care organizations for expert advice, controlled mixed interventions with experimental organizational arrangements, videoconferencing as an emerging technosocial service involving dialogue and empowerment of patients, and videoconferencing as a controlled intervention to improve administrative efficiency. The analysis across the clusters resulted in a proposal to add the following 4 D’s to the existing 10 E’s: (inter)-dependent, differentiated across services and along temporal lines, dynamic in terms of including novel elements for meeting incremental needs, and demanding in terms of making new challenges and dual results visible and needing fresh resources to meet those challenges. For a normative discussion about what eHealth should be according to authors’ conclusions, results suggested ethical, in that users interests should be respected, and not harmful in terms of increasing symptom burden.

**Conclusions:**

Services were enacted as dynamic, differentiated concerning content and considerations of quality and adaptive along temporal lines. They were made to work from an ongoing demand for fresh resources, making them interdependent. The 4 D’s—Dynamic, Differentiated, Demanding, and (inter) Dependent—serve as pragmatic add-ons to the “10 E’s of eHealth.” Questions concerning outcome of specified balances between standardization and customization in clinical settings should be addressed in future research along with the emerging dual character of outcome: services being considered both “good” and “bad.”

## Introduction

### Background

Videoconferencing has been used for half a century in health care. At its most sophisticated, it provides transmission of full-motion video and high-quality audio among multiple locations [1]. An early description of clinical videoconferencing involved real-time visits through cameras and televisions [2]. Such visits are still in clinical use and may include additional peripheral equipment such as stethoscopes, otoscopes, and derma scopes.

In 2010, Whitten et al [3] summarized the most popular areas for telemedicine through videoconferencing to be telepsychiatry [4], pediatric emergency consults [5], stroke diagnosis and treatment [6], teledialysis [7], and teledermatology [8].

Videoconferencing is part of electronic health (eHealth), and definitions and compositions of such services have developed along with technological refinement, changes in roles and responsibilities in health care and other contextual conditions. A 2005 systematic review identified 51 unique definitions of eHealth and concluded that it had become an accepted neologism despite the lack of clear, precise, agreed-upon definitions [9].

Challenges regarding the understanding of the notion, of use and nonuse, and the quality of such services have frequently been reported and can be connected with problems of determining what the intervention exactly consists of, as well as generalizable causes and effects due to the complexity of contexts [10]. In addition, earlier papers and reviews have demonstrated that videoconferencing often combines with customized organizational models and routines and specified human resources ranging from patients to family caregivers, nurses, and doctors, inhibiting different roles [11].

Moreover, complexities are demonstrated in studies considering videoconferencing as a component of interdependent relations among different technologies, the complexity of health care and the rituals and habits of patients and other stakeholders [12]. Furthermore, different purposes of videoconferencing have been described. In a 2014 systematic review of clinical use, 91% of included papers reported different clinical purposes, including diagnosis, treatment, counseling, and monitoring in a wide range of disciplines and settings [13].

The accounts above point to a complex field and the time seems to be right for obtaining contextualized knowledge of what kinds of services that work, for whom and under which circumstances. Granja et al also pointed this out in their recent systematic review of factors determining the success and failure of eHealth interventions [14]. They concluded that quality of care was most frequently mentioned as contributing to the success, and costs most frequently mentioned as contributing to the failure. In addition, they pointed to a critical need to perform in-depth studies of the workflow(s) that eHealth interventions support and to perceive the clinical processes involved.

This paper assumes that videoconferencing is interdependent with contexts and that workflow and quality consequently will be enacted and considered differently in different clinical processes. The paper provides an in-depth account of videoconferencing services, user patterns, and considerations of quality in different clinical practices.

The account is based on results of a critical-realist literature review, performed to explore what kinds of videoconferencing services were associated with what kinds of quality in different clinical processes, as presented in the academic literature [15].

We first present the results as a thick description of user patterns, by developing a narrative synthesis of comparable and contrasting services and considerations of quality. This synthesis is presented in clusters empirically ordered by similar technosocial configurations. Notably, this approach is grounded.

Second, we interpret the findings through an in-depth analysis [16]. We performed an exercise of reciprocal translation of the clusters and suggested and argued for common concepts and metaphors that are played out and can be applied to all clusters. This is a constructive analytical technique described under the theoretical umbrella of qualitative meta-synthesis [17].

Finally, after this exercise, we move on to discuss the concepts related to previous characteristics of eHealth. For this purpose, we rely on definitions and the “10 E’s of eHealth” as developed by Eysenbach [18]. The objective is to use our concepts as input for generative learning and further discussion of the concept of eHealth and considerations of quality.

In the following section, we provide an account of the critical-realist review, grounded perspective, concept of enactment, and steps in the qualitative meta-analysis as examples of research to obtain generative learning for the pragmatic adaptation of concepts.

We hope to get a deeper insight into prerequisites and impact in different health care practices [19]. Similarly, we hope to contribute to the knowledge of complexity and variety and, consequently, different understandings of eHealth [20].

This review can also be considered as an attempt to respond to a recent call for “new standards of research quality, namely (for example) rich theorizing, generative learning, and pragmatic adaptation to changing contexts in open systems characterized by dynamically changing interrelationships and tensions” [21].

In 2001, Gunther Eysenbach defined eHealth as a concept as follows: “In a broader sense, the term characterizes not only a technical development but also a state-of-mind, a way of thinking, an attitude, and a commitment for networked, global thinking, to improve health care locally, regionally, and worldwide using information and communication technology.” [18] This definition was also elaborated on through 10 characteristics, Eysenbach's 10 E’s of eHealth [18].

In a following series of publications, different characteristics were developed [22,23]. In 2015, the question “What Is eHealth: Time for an Update?” was posed [24].

### Theoretical Approaches to the Study

The paper refers to 2 different types of literature review, namely realist review and critical review [15,25]. The objectives also point to the specified features of grounded theory [16]. In the methods section, we elaborate the interconnection with grounded theory and the analytical perspective of meta-synthesis.

#### Realist Review and the Account of Diversity

Through realist review, we addressed the complexity of videoconferencing. Traditional methods of review may focus on measuring and reporting on generalizable program effectiveness; these often find that the evidence is mixed or conflicting, and provide little or no help to understand the specified features of services that worked or did not work in different contexts for different stakeholders or different purposes [25].

Realist review is a model of research synthesis designed to work with complex social interventions or programs, based on the emerging “realist” approach to evaluation. It provides an explanatory analysis aimed at discerning what works (descriptions of compositions of technologies, human resources, relations, and organizational arrangements) for whom (actors and roles), in what circumstances (clinical setting and socioeconomic setting if described), and in what respects (outcome measures and considerations of quality). Furthermore, a realist review often assesses how services work in terms of technological performance; of note, this element is not discussed in detail in our review.

#### Critical Review and Conceptual Innovation

A critical review goes beyond mere description of identiﬁed services and includes analysis and conceptual innovation [15]. Grant and Booth [15] argued that its product most typically manifests in a hypothesis or a model, not a categorical answer. While such a review serves to aggregate the literature on a topic, the interpretative elements, as in developing concepts, are necessarily subjective and the resulting product is the starting point for further discussion, not an endpoint. We pursue the development of metaphors or concepts to capture novel features that apply to all empirical clusters.

How can we shed light on the concept of eHealth through interpretations and conceptual constructs that apply to or emerge within all clusters? The meta-synthesis method described in the Methods section provides the strategy for such analyses. In addition, a critical review may also attempt to resolve competing schools of thought. As such, it may provide a “launch pad” for a new phase of conceptual development and subsequent testing. We will not address competing schools of thought but add-ons to existing concepts of eHealth.

For this purpose, we use the findings to compare and contrast with previous theoretical standpoints concerning eHealth as described in the 10 E’s. This is a deductive turn, and we relate our inductively derived concepts to the existing theory of eHealth.

## Methods

### Study Design

On September 15, 2015, we performed a systematic search for primary studies of videoconferencing in clinical practice. We searched through the National Center for Biotechnology Information, encompassing PubMed and PubMedCentral, using the terms “videoconferencing” and “clinical practices.” In addition, we included additional references from suggestions on the National Center for Biotechnology Information Web page or reference lists of papers identified in the original searches. This search was updated on May 15, 2018.

### Inclusion Criteria

The review included primary studies published in English between January 1, 2010, and May 15, 2018, which reportedly implemented clinical videoconferencing or Web conferences with the synchronous interaction between, at least, 2 geographical locations. Included in services were technological interventions, singular or as part of a composite service, and the paper should include a clear research objective or question with a clearly described method. Both qualitative and quantitative methods were included.

### Exclusion Criteria

Videoconferencing for educational or guideline development purposes and psychiatric or psychological interventions were excluded if these were not combined with somatic purposes. The reasons for excluding psychiatry and psychology were pragmatic, as the search yielded a large number of papers addressing such interventions. In addition, we excluded monitoring of vital signs and self-help apps if not combined with synchronous video or Web communications. Furthermore, feasibility and pilot studies were excluded.

### The Preferred Reporting Items for Systematic Reviews and Meta-Analyses Diagram

**Figure 1:**
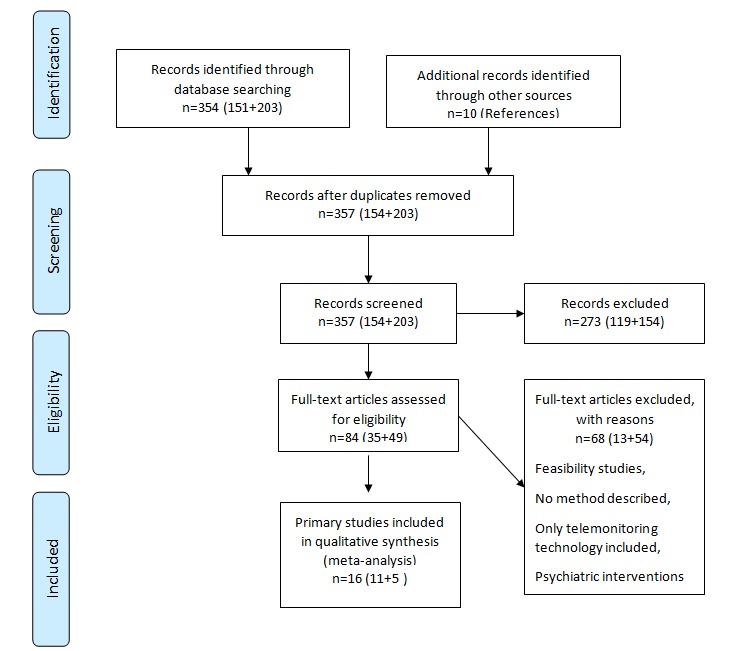
The PRISMA (Preferred Reporting Items for Systematic Reviews and Meta-Analyses) diagram.

The Preferred Reporting Items for Systematic Reviews and Meta-Analyses (PRISMA) diagram lists the steps of the inclusion and exclusion of papers (Figure 1).

### Descriptive Summary

An electronic form was developed and used to extract data from the included papers (see Multimedia Appendix 1). These data items were selected as follows: first author and title, year published, population and clinical area, description of the intervention, additional technologies included in the intervention, participatory or organizational arrangements, outcomes, goals, conclusion, challenges, suggestions for service improvements, and suggestions for further research. Multimedia Appendix 2 provides a table of results, created using Excel.

### Enactment of Clusters and Grounded Theory

The concept of enactment is used to suggest which practices of videoconferencing may play out differently through interdependencies [26,27]. A grounded perspective as described by Strauss and Corbin implies that empirical enactments should be the basis for deriving theoretical concepts, an inductive approach [16]. Thus, grounded theory is different from the deductive research model, that is, where the researcher chooses an existing theoretical framework (here the 10 E’s of eHealth) and collects data to show how the theory does or does not apply to the phenomenon under study.

We formed clusters by comparing and contrasting features responding to the realist review categories to specify the descriptive elements—what works and in what clinical setting. We coded for content, emerging themes, patterns, and novel ideas to also consider the 2 additional realist review themes, responding to what counts as good, for whom (actors and roles), and in what respects (outcome measures, considerations of quality, and suggestions for improvement). Furthermore, we used comparative methods involving open, axial, and selective coding of data where units of text (words, phrases, sentences, or paragraphs) were labeled, compared, and grouped [16].

### Meta-Synthesis and the Development of Concepts

A conceptual meta-synthesis is an interpretive, analytical technique that uses qualitative findings reported in previous studies as building blocks for gaining a deeper understanding of particular phenomena [17].

To perform a meta-synthesis, we used the clustered summary to interpret and suggest concepts pertaining to the descriptions and considerations to construct conceptual generalizations. As data were rereviewed, we grouped categories that formed the basis for suggesting the theoretical concepts.

Our meta-synthesis attempted to integrate results from a number of different but interrelated qualitative and quantitative studies. The technique has an interpretive, rather than aggregating, intent, in contrast to the meta-analysis of quantitative studies.

### Proposing New Concepts for Generative Learning: the “10 E’s of eHealth”

Within the clustered summaries, we first very briefly identified examples to provide connections with Eysenbach's 10 E’s. Through this strategy, we showed how the services we investigated aligned with or accommodated the 10 E’s. But the main focus of the analysis was to propose and argue for new concepts to contribute to generative learning, that is, what might be added to the 10 E’s from our findings and interpretations? This implies that we suggest concepts for pragmatic adaptation to the existing 10 E’s.

## Results

### Descriptive Summary

The 16 included primary studies covered 13 clinical areas, some of which were overlapping—chronic neurological care, dermatology and heart disease, stroke, neonatal care, primary health care, neuromuscular disease, pathology, palliative care, chronic care [hypertension, chronic obstructive pulmonary disease (COPD), and cardiovascular diseases combined], primary headache, pediatric telegenetics, rehabilitation of total knee replacement, and geriatric rehabilitation. In addition, we included outpatient triage. Studies reporting both quantitative and qualitative methods were included.

We have provided an overview of the data extracted from each of the included primary studies by listing the first author and title, year published, patient populations, clinical area, technosocial interventions, outcome measures, results, conclusion, any suggestions for improvement of the intervention and further research as an appendix (Multimedia Appendix 1).

We derived 4 broad clusters based on similarities in technosocial compositions. The clusters are as follows: videoconferencing as a controlled technological intervention within existing health care organizations for expert advice, including 5 papers [28-32]; controlled mixed interventions with experimental organizational arrangements, including 2 papers [33,34]; videoconferencing as an emerging technosocial service involving dialogue and empowerment of patients, including 8 papers [35-42]; and videoconferencing as a controlled intervention to improve the administrative efficiency, including 1 paper [43].

We suggested adding the 4 D’s to Eysenbach's 10 E’s of eHealth — eHealth is also (inter) dependent, differentiated across services and along temporal lines, dynamic in terms of incrementally including novel elements for meeting needs, and demanding in terms of making new challenges and dualistic results visible, and being in need of fresh resources to meet emerging challenges.

For a normative consideration, what eHealth also should be according to authors’ conclusions, we added ethical, in that users’ interests should be respected, and not harmful in terms of increasing the symptom burden.

### Clusters, Characteristics, and Suggestions for Improvement

The 4 clusters were organized and elaborated according to the categories of realist review—what works (compositions of technologies, human resources, relations, and organizational arrangements), for whom (actors and roles), and in what circumstances (clinical setting and socioeconomic setting if described). This account makes up the descriptive part of the results, and we have clustered services that are comparable concerning the composition. We further considered the outcome and quality by assessing authors’ proposals for improvement of services, to respond to the question of how services are considered and what counts as good (Multimedia Appendix 1).

### Videoconferencing as a Controlled Expert Intervention Within Existing Organizational Arrangements

#### What Works, for Whom, in Which Clinical Area, and With Which Outcome: What eHealth Is

In chronic neurological care, videoconferencing was used in follow-ups from specialists to veterans living in rural areas, after an initial complete historical and neurological examination. Patients were highly satisfied with the outcomes of convenience and quality [28].

In dermatology and heart disease, specialist care was delivered in a primary health care center in rural areas. A majority of patients considered the services as a positive experience, particularly, as specialist visits were expensive [29]. In the UK National Health Service, a regional telestroke network comprising 7 district general hospitals delivered thrombolysis with effective management, access, and safety as an outcome; this was conditionally successful [30]. In medical treatment for a primary headache, the service consisted of face-to-face interviews, examination, cranial magnetic resonance imaging, and electroencephalogram, a discussion between 2 physicians at different locations, and prescription of medication, all through data and video transmission. The network demonstrated safe and efficacious delivery of services [31].

In weekly specialist palliative care, teleconsultations for patients with advanced cancer were compared with “care as usual” [32]. Furthermore, the patient-experienced symptom burden was discussed.

#### Considerations and Proposals for Improvement of Services’ Quality

During assessments, complexities appeared that problematized generalizable conclusions for all services, and further success seemed to rely on the customization of certain aspects. For instance, results on the quality were narrowed to apply to more specified conditions in chronic neurological care [28], going through several encounters were considered a prerequisite to improving patients’ experience of safety in dermatology and heart disease [29], and variability of success between local sites was reported in thrombolysis services [30]. In addition, further success turned out to depend on additional resources involving expertise from nurses or technicians [28] and social and human relations like collaboration and confidence [29]. Telemedicine did not necessarily lead to a better quality of advanced cancer care. Indeed, the use of telemedicine created a situation in which patients experienced a higher symptom burden, despite high degrees of satisfaction. Authors proposed further research on ways to optimize multidisciplinary care by teleconsultations and to decide appropriate timing and frequency of palliative care teleconsultations [32].

eHealth was enacted as conditionally successful and partly with contrasting results as in palliative care. eHealth was demanding new resources and customization of organizational, diagnostic, and ethical aspects. The balance between standardization or customization was played out as a challenge, as was the balance between fixed or dynamic and adaptive solutions.

### Mixed Services With Novel Organizational Approaches for Expert Advice

#### What Works, for Whom, in Which Clinical Area, and With Which Outcome?

A novel approach in pediatric telegenetic services was described, comprising a geneticist, pediatrician, and genetic counselor team [33]. A telegenetics clinic offered a viable solution to providing competent and convenient access to a geneticist for patients in chronically underserved regions. For telerehabilitation after total knee replacement in Italy, a cost-effectiveness and cost-utility analysis was performed of a mixed telerehabilitation-standard rehabilitation program compared with usual care. Cost savings were documented [34].

##### Considerations and Proposals for Improvement of Services’ Quality

Further studies were requested to define the outcomes of pediatric genetic evaluations better and specify which outcomes most appropriately helped to determine the satisfaction and efficacy of telegenetics evaluations compared with in-person genetic evaluations [33]. Uncertainty related to costs and long-term clinical outcomes raised important topics for future research in the rehabilitation study [34]. Moreover, in this cluster, more specified knowledge of outcomes, that is, the quest for more detailed and customized differentiation of services to understand quality was communicated. The dynamic adaptation of services was requested following the uncertainty of long-term effects.

### Videoconferencing as Part of a Composite Service Involving Dialogue and Empowerment of Patients

#### What Works, for Whom, in Which Clinical Area, and With Which Outcome?

Birth delivery patterns in neonatal care in a state network was addressed in weekly educational videoconferences to establish guidelines for obstetrical, neonatal, and pediatric care at rural hospitals and telenursery sites [35]. In addition, the network maintained a 24-hour call center staffed by experienced nurses who provided case management for patients and their physicians across the state, including appropriate transfer of high-risk patients to regional perinatal centers associated with high-quality delivery. The service was considered successful on all outcome measures, particularly for rural and underserved populations.

In neuromuscular diseases, 2 different telecare protocols were followed depending on the severity of the patient’s condition [36]. One group combined videoconferencing with face-to-face consultation once a month to see the patient in person and check the equipment, whereas the other maintained face-to-face consultation. The system was effective for home treatment and reduced the need for hospital admissions.

The Eastern Quebec Telepathology Network included a macroscopy station and 2 videoconferencing devices equipped with drawing tablets. The assessment considered the real-time evaluation of the concordance rate, turnaround time, and effects of telepathology on health care professionals, patients, organization, and delivery of care, from 3 years’ experience [37]. The service reduced isolation and insecurity among pathologists working alone.

Interdisciplinary team group videoconference meetings every second week with the involvement of informal caregivers was as part of a total home-care program for hospice patients [38]. The role of technology was a mixed experience, and short interaction length was sometimes frustrating, but most caregivers reported feeling part of the team and were positive about the technology experience. Caregivers had positive relationships with hospice staff, felt involved in decision making, and got questions answered.

“Tile-Ippokratis,” was an integrated platform providing low-cost eHealth services to elderly patients with chronic diseases and postsurgery patients with hypertension, COPD, and cardiovascular disease [39]. The platform reduced hospital admissions, provided safety, self-management, and quality of life. Users from both server and client units expressed positivity on the interface and data entering procedures of the devices used.

In outpatient palliative care, videoconferencing facilitated empathic patient–professional relationships [40]. Owing to rapid technological developments, the following 2 teleconsultation devices were used during the study: a desktop computer and an iPad 2. Results were focused attention and listening, the empathic engagement between patients and palliative care specialists, and an opportunity for patients to co-design their own care within the comfort of their homes. The potential of teleconsultations jeopardizing privacy was reported.

Another study addressed the same service with a focus on professional collaboration [41]. Synchronous audiovisual teleconsultation between a hospital-based specialist palliative care team and home-based palliative care patients was added to an existing community care model. The introduction of specialist teleconsultation in palliative home care supported multidisciplinary care.

For older rehabilitation, patients’ telerehabilitation by weekly home visits was combined with monitoring of activity data and videoconference via iPad [42]. Positive outcomes were reported in terms of the experience, activity levels, fitness, functioning, and well-being.

#### Considerations and Proposals for Improvement of Services

In neonatal care, longitudinal knowledge on system-oriented changes was requested [35]. In neuromuscular diseases, adaptive organizational procedures and face-to-face consultations should be more developed to improve services [36]. In telepathology, a strong communication plan and highly coordinated efforts among surgeons, pathologists, stakeholders, laboratory staff, and biomedical, administrative, and information technology support teams working in different sites were considered crucial for further development [37]. In elderly care, palliative and hospice care, familiarization of technologies, more frequent meeting times, and additional training for family caregivers were needed [38,40]. In addition, further suggestions included the following: to enable local health care systems and different group populations to be familiarized with and use mature technological solutions [40,41], to address emerging ethical questions [40], the need to familiarize with tripartite consultations, and to adapt primary care physicians’ routines to *ad hoc* meetings [37]. Older rehabilitation patients valued face-to-face contact with their therapist, even when they are very positive about their telerehabilitation experience. This perception of telerehabilitation as complementary rather than a substitute for in-person care indicates that an ideal telerehabilitation service would continue to provide traditional therapy options by interspersing face-to-face contact with distance therapy wherever possible [42].

eHealth was enacted as highly heterogeneous and interdependent concerning composition, roles, and outcome in this cluster. In addition, dynamic adaptation is a keyword that captures services. Services were composed as bespoke, tailored, and interdependent of a variety of human and organizational components.

### Videoconferencing as a Means for Administrative Efficiency Improvement

#### What Works for Whom and in Which Clinical Area?

Video telehealth on outpatient clinic triage nurse workflow consisted of the experimental use of personal computer-based videoconferencing software between home and an outpatient clinic triage office to communicate health questions and concerns that would otherwise be communicated by telephone [43]. For this service, technologies were considered unpredictable, as disrupting existing and well-working routines and dependent on the increased human effort.

#### Considerations and Proposals for Improvement of Services

Authors referred to a proposal from the involved to extend the scope of involved professionals for assessing a broader spectrum of components to conclude about the effectiveness. In that respect, the service turned out to be more complex than had been assumed before the investigation. This service was disruptive of existing services that were considered as well functioning.

### Summary

In chronic neurology, dermatology, and heart disease, thrombolysis for stroke, and palliative home care, videoconferencing was addressed as a singular technological intervention within existing, but varying, organizational arrangements where expert roles were clearly defined. The characteristics were evidence-based advice between specialists and patients, specialists and primary care doctors, and a specialist center and rural hospitals. Success on outcome measures of the effectiveness, convenience, and safety for patients and clinical quality was described. The authors asked for changes in target groups and additional human professional resources for improvement or stabilization. In addition, the authors suggested the appropriate timing and frequency of palliative care teleconsultations, which had shown both positive and negative effects.

In telegenetic services and rehabilitation after total knee replacement, mixed services and novel organizational arrangements were described. Cost savings and the viability of services were demonstrated, and the authors asked for better specification of appropriate outcome and improved knowledge of long-term clinical effects.

In neonatal care, neuromuscular diseases, dermatology and heart disease, telepathology, palliative care, chronic diseases of hypertension, COPD, and cardiovascular diseases, novel organizational arrangements and composite services were described; they were diverse, outreaching, and in need to adapt to contexts and specified challenges. All services were described as conditionally successful but were considered both positive and negative in palliative care services, raising new ethical and relational questions to be addressed for future improvements. It became apparent that videoconferencing posed certain incremental demands for new resources in terms of improved technological features, changes in organizational arrangements, and new professional and ethical efforts. Elderly rehabilitation patients value face-to-face contact with their therapist, even when they are very positive about their telerehabilitation experience.

In triage, videoconferencing was considered as unpredictable and as disrupting existing and well-working routines. Success on the effectiveness was dependent on the increased human effort and extension of involved professionals.

## Discussion

### Generative Learning and Conceptual Innovations: Adding the 4 D’s to the 10 E’s of eHealth

We now present the reciprocal translation of the clusters to reach a synthesis by suggesting and arguing for concepts. To further discuss the concepts, we first connect some of the findings to the 10 E’s of eHealth and 2 normative statements on what eHealth should be.

### The 10 E’s of eHealth and Some Characteristics From the Results

Below, Eysenbach's 10 E’s of eHealth are listed, along with some characteristics from the results:

1. *Efficiency*: all services in clusters 1 and 4 responded to this characteristic either as goals or as achievements. In clusters 2 and 3, the efficiency was less emphasized.
2. *Enhancing the quality of care*: all services responded to this characteristic as goals and partly as achievements, but the quality was considered very heterogeneously and was ambiguous for palliative care.
3. *Evidence-based*: videoconferencing was partly established on the basis of evidence but also as emerging services, taking one step at a time. Evidence production is a returning challenge in the field as services are adaptive and not always controlled interventions. This is an important issue, which is not addressed in this paper. Perhaps, “early procurement with evidence generation” using participatory approaches as proposed in Health Technology Assessment (HTA) literature is a way to proceed [44].
4. *Empowerment of consumers and patients*: this was a dedicated goal partly for cluster 2 and for all services in cluster 3.
5. *Encouragement of a new relationship between patients and health professionals toward a true partnership*: this was explicitly stated as a dedicated goal for family caregivers and partly for patients in palliative care.
6. *Education of physicians through online sources*: as described for the telepathology network.
7. *Enabling information exchange and communication in a standardized way between health care establishments*: all services included this as a goal and partly as a characteristic.
8. *Extending the scope of health care beyond its conventional boundaries*: as described for the home care services in palliative care.
9. *Ethics*: eHealth involves new forms of the patient–physician interaction and poses new challenges and threats to ethical issues such as online professional practice, informed consent, privacy, and equity issues; this was addressed especially in palliative care.
10. *Equity*: to make health care more equitable is one promise of eHealth, but at the same time, there is a considerable threat that eHealth may deepen the gap between the “haves” and “have-nots.” Equity was addressed in chronic neurological care, dermatology, and heart disease for thrombolysis, neonatal care, and telepathology.

In addition to these 10 essential E’s, Eysenbach [18] also proposed that eHealth should be:

1. *Easy-to-use*: Our results indicate that this is still a goal and there is still work to bedone, as improvements are requested. Easier use was specially requested for the triage workflow.
2. *Entertaining (no one will use something that is boring!) and exciting*: this subject was not addressed in any of the services, but videoconferencing was considered to interrupt existing and well-working routines in triage.

### Adding the 4 D’s to the Concept of eHealth

The quests for different new resources and organizational arrangements revealed videoconferencing as dependent, demanding, and in need to customize to reach goals.

The following 4 D’s can be interpreted as generalizable concepts from our review and serve as input to generative learning: (inter) Dependent; Differentiated across services and temporal lines; Dynamic; and Demanding. The 4 D’s are not easily distinguished as they are also interdependent. We organized the discussion by first coupling the 2 D’s (inter)-dependency and differentiated, and then demanding and dynamic. The former 2 refer to the existing characteristics and the latter to assessments of the existing and future prospects. We interpret these 4 D’s as enacted both within and across clusters and have combined clusters 1 and 4 and 2 and 3 for substantiation. In addition, we propose that eHealth should be:

1. Ethical in that users’ interests should be respected
2. Not harmful by increasing the symptom burden

#### (Inter) Dependent and Differentiated Services in Clusters 1 and 4

All papers in clusters 1 and 4 [28-32] and [43] were addressed as relatively singular and controlled interventions addressing effective management or coordination. In addition, standardized communication and decision support were expected to improve the clinical quality and patient satisfaction. Considerations about what was needed to sustain services pointed to additional human and organizational resources and more specified solutions. These considerations point to dependent services.

Differences were displayed in the characteristics of the conditions that were considered, as 2 papers addressed singular clinical conditions [28,30], 1 multiple conditions [29], and 1 triage and workflow [43]. In addition, 3 of the 4 papers described services within fixed but different organizational structures—a specialist (center) to local or primary care professionals or patients and with fixed location of the equipment [28-30]. One paper described a network sharing a “virtual consultations room” and differences in results of the intervention for different local sites. Thus, stakeholders indicated dependency and differentiation determined by local variables to explain results [30].

These papers, at the outset considering videoconferencing as a relatively singular intervention, by implication concluded that each service was composite, as well as differentiated across services. Likewise, all services were interdependent, as the inclusion of specific organizational and human resources were considered crucial for continued success. Diversity across services and temporal lines, as well as interdependencies among technological, socioeconomic, and human components, were enacted.

#### Dynamic and Demanding Services in Clusters 1 and 4

One paper reported negative effects in terms of reduced efficiency. The authors suggested that to conclude, additional organizational aspects like physician time use should be considered. The services played out as dynamic and emerging. For the service to succeed, additional professionals were demanded, as well as further investigations of the impact on the overall clinic workflow [43]. Three papers concluded that the service provided was valuable, but reported the need for additional professional nurses or a health technician to improve the services [28-30]. One reported the need to address issues of increased symptom burden in palliative care [32]. Their assessment pointed both to the interdependency of human resources and demanding and dynamic services. All authors suggested that more knowledge of human (diverse patient populations and competence) and new organizational arrangements was necessary for improvement and more insight.

Videoconferencing was enacted as demanding and dynamic in terms of pressure to incrementally increase human and organizational resources for continued improvement, as well as addressing issues of the increased symptom burden.

#### (Inter) Dependent and Differentiated Services in Clusters 2 and 3

Common features deriving from this cluster aligned with the former discussions. A closer look at the papers substantiates composite, diverse, and interdependent services by the inclusion of new outreach and additional technological components. Five papers described services provided to patients’ homes [36,38-41]. One of these reported advantages of introducing tablet computers to palliative care patients at home [40]. Two papers included monitoring technologies in homes [36,39].

Regarding outcome, the majority reported multiple outcome that substantiates differences across services. Two papers pointed to information exchange, standardized communication, reduced hospital admissions, reduced home visits, and clinical impact [36,37], and one described transcending of institutional walls, clinical quality, and technologized but intimate patient and palliative care specialists’ relationships. The authors further considered the opportunity for patients to co-design their own care within the comfort of their homes [40]. While one addressed economic issues (cost-effectiveness or reduction) and quality of life [39], another mentioned retention and recruitment of surgeons to remote hospitals and reduced isolation [37]. In addition, one paper addressed changes in delivery patterns with the intention to refer high-risk newborns from local to regional perinatal centers [35]. One paper addressed new relations between participants and shared decision making by stimulating the integration of primary and specialist palliative care [41].

Technologies were interdependent with peripheral technological equipment involving tablet and monitoring devices and with relational and organizational components as expressed through outreach to patients’ homes, shared decision making, and intimate relationships.

#### Dynamic and Demanding Services in Clusters 2 and 3

The key to further success of telepathology was to maintain and develop a strong communication plan and highly coordinated effort among surgeons, pathologists, stakeholders, laboratory staff and biomedical, administrative, and information technology support teams working in different sites [37]; this key indicated both interdependencies, dynamic development and a demand for additional efforts to obtain objectives.

In palliative care, new ethical questions appeared, which needed attention to meet goals [38,40,41]. The need for additional education, consultation, and guideline dissemination was claimed in neonatal intensive care [35]. In these clusters, the normative aspects also played out—eHealth should be ethical and not serve to increase symptom burden.

#### Summary

The analysis showed that services were highly interdependent, composite, and diverse across services and temporal lines, as compositions of videoconferencing and peripheral equipment, organizational and human resources, purposes and goals differed between them. They were also demanding in terms of pressure to incrementally increase or change human and organizational resources for continued improvement. The services were dynamic in that new developments were anticipated for continuous development, ranging from dedicated organizational components to strong communication plans involving all stakeholders. Clearly, the 4 D’s were highly recognizable and striking in the clusters.

### Limitations

To provide a complete account of videoconferencing services in clinical practices in the period covered, the review has limitations in the number of databases that were searched, the search terms, and the 7-year temporal limitation; this was, however, a deliberate decision because we wanted a quick view of recent service configurations. It can be expected, however, that broader search criteria, more databases, and a longer time span would provide even more differentiation and heterogeneity. In that sense, this review provides an informative content that responds to the ideas of realist review. Furthermore, the review was limited in that we did not provide a detailed account of working mechanisms, the how, of different practices.

### Comparison With Prior Work and Pragmatic Implications

A systematic review from 2015 addressed technical characteristics of video consultations and ways these had changed over time because of the rapid advancement of information and communication technology [45]. The most widely used hardware described for videoconferencing was dedicated videoconference codecs and personal computers (desktop, laptop, or notebook). In addition, the review reported that the usage of mobile or smartphones for clinical videoconferencing started in 2005 and concluded that this could be an early indication of a marked change from fixed, dedicated hardware (eg, codecs or personal computers) toward ubiquitous devices (eg, smartphones or tablets). The shift to tablets in palliative care supports this change.

In a systematic review of telepathology services from 2016, Farahani et al concluded that mechanisms of success for international telepathology services were efficient workflow, dedicated information technology staff, continuous maintenance, financial incentives, ensuring that all stakeholders were satisfied, and value-added clinical benefit to patient care [46]. Our review has contributed to in-depth knowledge of aspects of workflow, dedicated staff, and value-added clinical benefit in specified clinical settings. What counted as an efficient workflow, dedicated staff, ensuring satisfaction and value-added clinical benefit was highly heterogeneous.

### Conclusions

Videoconferencing in clinical practices was enacted as heterogeneous and with bespoke prerequisites and implications. Services were dynamic, differentiated concerning the content and considerations of quality, and adaptive along temporal lines. They were made to work from ongoing demand for fresh resources, making them interdependent. The 4 D’s—Dynamic, Differentiated, Demanding, and (inter) Dependent—serve as a pragmatic add-on to Eysenbach's 10 E’s of eHealth. Questions concerning the outcome of specified balances between standardization and customization in clinical settings should be addressed in future research, as well as the emerging dual character of outcome—services being considered both “good” and “bad.”

## Appendix

Multimedia Appendix 1 Electronic proforma.

Multimedia Appendix 2 Included primary studies.

## Abbreviations

COPD: chronic obstructive pulmonary disease
eHealth: electronic health
HTA: health technology assessment

## References

[1] TechTarget (2016). SearchUnified Communications.

[2] Wittson CL, Affleck DC, Johnson V (1961). Two-way television in group therapy. Ment Hosp.

[3] Whitten P, Holtz B, Laplante C (2010). Telemedicine: What have we learned?. Appl Clin Inform.

[4] Hilty D, Yellowlees P, Cobb H, Bourgeois J, Neufeld Jonathan D, Nesbitt T (2006). Models of telepsychiatric consultation--liaison service to rural primary care. Psychosomatics.

[5] Heath B, Salerno R, Hopkins A, Hertzig J, Caputo M (2009). Pediatric critical care telemedicine in rural underserved emergency departments. Pediatr Crit Care Med.

[6] Levine SM, Gorman M (1999). "Telestroke": the application of telemedicine for stroke. Stroke.

[7] Whitten PL, Buis Lorraine (2008). Use of telemedicine for haemodialysis: perceptions of patients and health-care providers, and clinical effects. J Telemed Telecare.

[8] Burdick A (2007). Teledermatology: extending specialty care beyond borders. Arch Dermatol.

[9] Oh H, Rizo C, Enkin M, Jadad A (2005). What is eHealth (3): a systematic review of published definitions. J Med Internet Res.

[10] Greenhalgh TJ, Russell Jill (2010). Why do evaluations of eHealth programs fail? An alternative set of guiding principles. PLoS Med.

[11] May C (2006). A rational model for assessing and evaluating complex interventions in health care. BMC Health Serv Res.

[12] Rip A, Kulve H, Fisher E, Selin C, Wetmore JM (2008). Constructive Technology Assessment and Socio-Technical Scenarios. Presenting Futures: The Yearbook of Nanotechnology in Society, Vol 1.

[13] Fatehi F, Armfield NR, Dimitrijevic M, Gray LC (2014). Clinical applications of videoconferencing: a scoping review of the literature for the period 2002-2012. J Telemed Telecare.

[14] Granja C, Janssen W, Johansen MA (2018). Factors Determining the Success and Failure of eHealth Interventions: Systematic Review of the Literature. J Med Internet Res.

[15] Grant M, Booth A (2009). A typology of reviews: an analysis of 14 review types and associated methodologies. Health Info Libr J.

[16] Strauss A, Corbin JM (2015). Basics of Qualitative Research: Techniques and Procedures for Developing Grounded Theory.

[17] Walsh D, Downe S (2005). Meta-synthesis method for qualitative research: a literature review. J Adv Nurs.

[18] Eysenbach G (2001). What is e-health?. J Med Internet Res.

[19] van Gemert-Pijnen JE, Nijland N, van Limburg M, Ossebaard HC, Kelders SM, Eysenbach G, Seydel ER (2011). A holistic framework to improve the uptake and impact of eHealth technologies. J Med Internet Res.

[20] Pagliari C, Sloan D, Gregor P, Sullivan F, Detmer D, Kahan JP, Oortwijn W, MacGillivray S (2005). What is eHealth (4): a scoping exercise to map the field. J Med Internet Res.

[21] Greenhalgh Trisha, Papoutsi Chrysanthi (2018). Studying complexity in health services research: desperately seeking an overdue paradigm shift. BMC Med.

[22] Jones R, Rogers R, Roberts J, Callaghan L, Lindsey L, Campbell J, Thorogood M, Wright G, Gaunt N, Hanks C, Williamson GR (2005). What is eHealth (5): a research agenda for eHealth through stakeholder consultation and policy context review. J Med Internet Res.

[23] Ahern DK, Kreslake JM, Phalen JM (2006). What is eHealth (6): perspectives on the evolution of eHealth research. J Med Internet Res.

[24] Boogerd EA, Arts T, Engelen LJ, van de Belt T (2015). "What Is eHealth": Time for An Update?. JMIR Res Protoc.

[25] Pawson R, Greenhalgh T, Harvey G, Walshe K (2005). Realist review–a new method of systematic review designed for complex policy interventions. J Health Serv Res Policy.

[26] Law J (2004). After method: Mess in social science research.

[27] Law J, Urry J (2011). Enacting the social. Economy and Society.

[28] Davis LE, Coleman J, Harnar J, King MK (2014). Teleneurology: successful delivery of chronic neurologic care to 354 patients living remotely in a rural state. Telemed J E Health.

[29] Johansson A, Lindberg I, Söderberg S (2014). Patients' Experiences with Specialist Care via Video Consultation in Primary Healthcare in Rural Areas. Int J Telemed Appl.

[30] Agarwal S, Day DJ, Sibson L, Barry PJ, Collas D, Metcalf K, Cotter PE, Guyler P, O'Brien EW, O'Brien A, O'Kane D, Owusu-Agyei P, Phillips P, Shekhar R, Warburton EA (2014). Thrombolysis delivery by a regional telestroke network--experience from the U.K. National Health Service. J Am Heart Assoc.

[31] Akiyama H, Hasegawa Y (2018). A trial case of medical treatment for primary headache using telemedicine. Medicine (Baltimore).

[32] Hoek PD, Schers HJ, Bronkhorst EM, Vissers KCP, Hasselaar JGJ (2017). The effect of weekly specialist palliative care teleconsultations in patients with advanced cancer -a randomized clinical trial. BMC Med.

[33] Kubendran S, Sivamurthy S, Schaefer G (2017). A novel approach in pediatric telegenetic services: geneticist, pediatrician and genetic counselor team. Genet Med.

[34] Fusco F, Turchetti G (2016). Telerehabilitation after total knee replacement in Italy: cost-effectiveness and cost-utility analysis of a mixed telerehabilitation-standard rehabilitation programme compared with usual care. BMJ Open.

[35] Hall R, Hall-Barrow J, Garcia-Rill E (2010). Neonatal regionalization through telemedicine using a community-based research and education core facility. Ethn Dis.

[36] Zamarrón C, Morete E, González F (2014). Telemedicine system for the care of patients with neuromuscular disease and chronic respiratory failure. Arch Med Sci.

[37] Têtu B, Perron E, Louahlia S, Paré G, Trudel M, Meyer J (2014). The Eastern Québec Telepathology Network: a three-year experience of clinical diagnostic services. Diagn Pathol.

[38] Oliver DP, Albright DL, Kruse RL, Wittenberg-Lyles E, Washington K, Demiris G (2014). Caregiver evaluation of the ACTIVE intervention: "it was like we were sitting at the table with everyone". Am J Hosp Palliat Care.

[39] Papadopoulos Homer (2010). Tile-ippokratis: the experience of an ehealth platform for the provision of health care services in the island of chios and cyprus. Int J Telemed Appl.

[40] van Gurp J, van Selm M, Vissers K, van Leeuwen E, Hasselaar J (2015). How outpatient palliative care teleconsultation facilitates empathic patient-professional relationships: a qualitative study. PLoS One.

[41] van Gurp J, van Selm M, van Leeuwen E, Vissers K, Hasselaar J (2016). Teleconsultation for integrated palliative care at home: A qualitative study. Palliat Med.

[42] Shulver W, Killington M, Morris C, Crotty M (2017). 'Well, if the kids can do it, I can do it': older rehabilitation patients' experiences of telerehabilitation. Health Expect.

[43] Cady RG, Finkelstein SM (2013). Mixed-methods approach for measuring the impact of video telehealth on outpatient clinic triage nurse workflow. Comput Inform Nurs.

[44] Facey K, Henshall C, Sampietro-Colom L, Thomas S (2015). Improving the effectiveness and efficiency of evidence production for health technology assessment. Int J Technol Assess Health Care.

[45] Fatehi F, Armfield NR, Dimitrijevic M, Gray LC (2015). Technical aspects of clinical videoconferencing: a large scale review of the literature. J Telemed Telecare.

[46] Farahani N, Riben M, Evans AJ, Pantanowitz L (2016). International Telepathology: Promises and Pitfalls. Pathobiology.

[47] Bergmo T, Ekeland AG (2013). Modelling VC cooperation: conditions, mechanisms and outcomes – a multi methodological study.

[48] Ekeland AG (2016). Health Technology Assessment (HTA) for ICT.

